# Pressure‐Dependent Aromatic Ring Flips Reveal Variable Transition‐State Volume and Compressibility Among Structural Regions of BPTI

**DOI:** 10.1002/cbic.70442

**Published:** 2026-06-30

**Authors:** Matthias Dreydoppel, Mikael Akke, Ulrich Weininger

**Affiliations:** ^1^ Institute of Physics, Biophysics Martin‐Luther‐University Halle‐Wittenberg Halle (Saale) Germany; ^2^ Division of Biophysical Chemistry Center for Molecular Protein Science Department of Chemistry Lund University Lund Sweden

**Keywords:** biophysics, high‐pressure NMR, NMR spectroscopy, protein dynamics, protein volume fluctuations, proteins, relaxation dispersion

## Abstract

Conformational transitions are critical for protein function and often involve substantial rearrangements of the interior packing of amino acid residues. Rotations of aromatic side chains—so called “ring flips”—require transient expansion of the surrounding protein core and represent hallmark examples of transient conformational fluctuations. We recently demonstrated that activation volume and isothermal compressibility provide unique insights into ring flip transition states. Here, we report transition‐state compressibilities for four aromatic side chains in bovine pancreatic trypsin inhibitor (BPTI). The compressibilities vary widely, ranging from values typical of unfolded proteins to those of the fully folded state. Notably, transition‐state compressibility correlates negatively with activation energy, indicating that the likelihood of a ring flip scales with the degree of expansion in the transition state. Our results show that different structural regions, even within a small protein such as BPTI, display distinct dynamic transitions and volume fluctuations, reflecting varying extents of structural rearrangement along the transition pathways. Thus, isothermal compressibility emerges as a powerful parameter yielding insights into the relationships between ground‐state structure and the transition‐state expansion during conformational transitions.

## Graphical abstract

Pressure‐dependent NMR studies of ring flip dynamics reveal an inverse relationship between the activation energy and the activation volume, as well as the isothermal compressibility of the transition state. The results show how side‐chain packing density in the ground and transition states might affect protein dynamics.

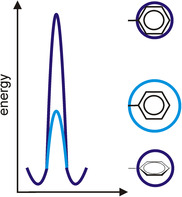

## 1 Introduction

Proteins are dynamic molecules that undergo transitions between alternative conformations that are essential for their biological function [1, 3]. Such functionally relevant protein dynamics require collective movement of many atoms, which often takes place on slow timescales (μs to ms). Aromatic ring flips—180° rotations of the aromatic ring around the imaginary C_β_‐C_γ_‐C_ζ_ axis (equivalently, 180° changes in the *χ*_2_ dihedral angle) in Phe and Tyr side chains—are a hallmark of protein dynamics [4, 5]. The discovery of ring flips in the mid‐1970s [6, 7] fundamentally transformed our understanding of proteins, revealing them to be inherently dynamic rather than static molecules.

Aromatic residues are overrepresented in protein binding sites and form an integral part of the hydrophobic core by contributing roughly 25% of the volume on average. For an aromatic ring flip to occur, the surroundings must undergo concerted motions that create sufficient void volume around the ring. Because aromatic residues are often located throughout the protein structure, they serve as useful probes for studying protein dynamics in the context of different structural and functional environments or specific interactions, like aromatic–aromatic pair interactions [8, 9] or interactions with cations [10] and sulfur atoms [11].

**FIGURE 1:**
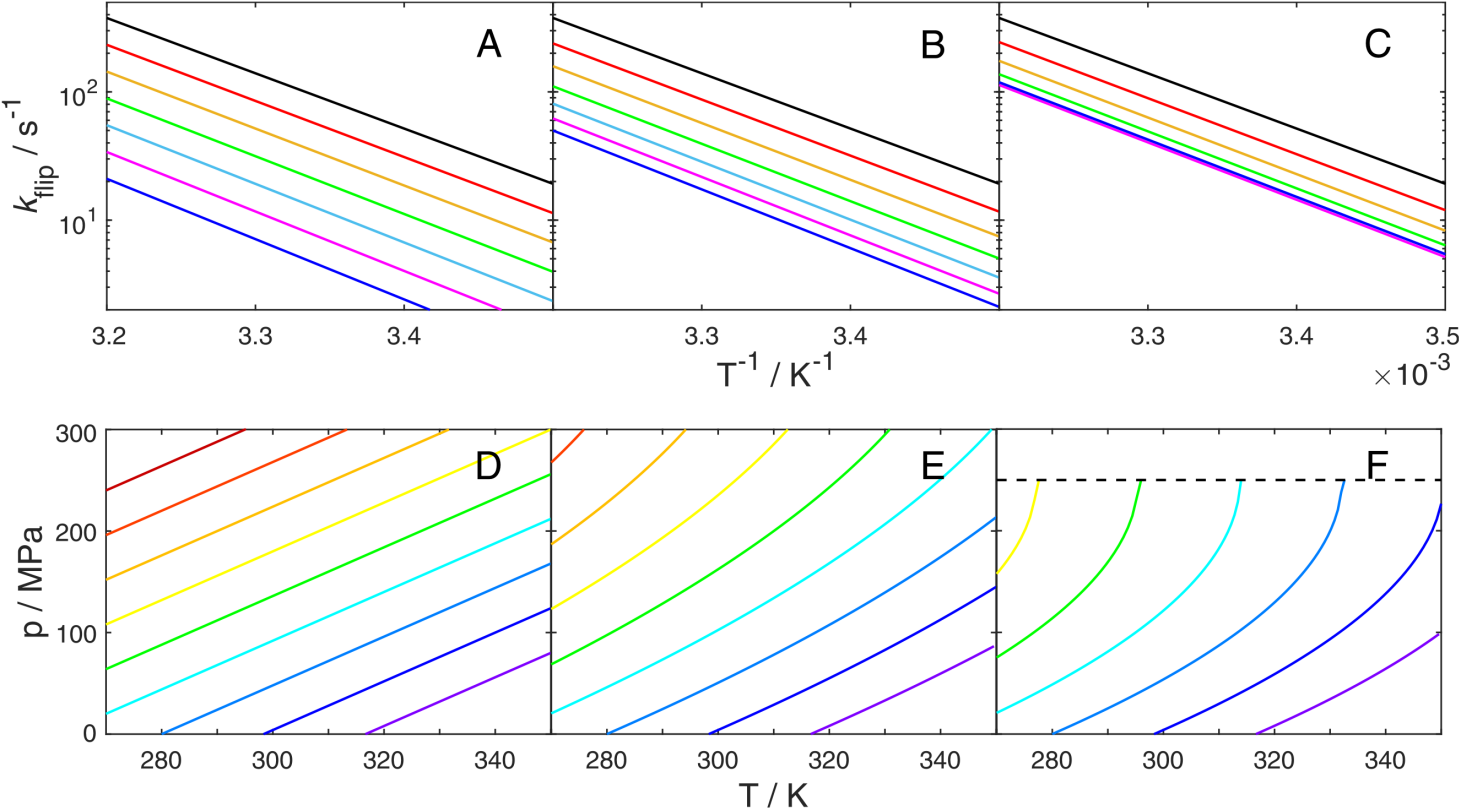
Theoretical temperature and pressure dependence of the ring flip rate (A–C) and Gibbs free energy of activation (D–F). *k*_flip_ is plotted as a function of 1/T (A–C) at pressures of 0.1 MPa (black), 50 MPa (red), 100 MPa (magenta), 150 MPa (green), 200 MPa (light blue), 250 MPa (magenta), and 300 MPa (dark blue). (D–F) Contour plots: constant values of the Gibbs free energy of activation are plotted as colored contour lines with Δ^‡^ *G* ranging equidistantly from 61 kJ mol^−^^1^ (violet) to 70 kJ mol^−1^ (red). Calculated using Equation (2) with Δ^‡^ *H* = 80 kJ mol^−1^, Δ^‡^ *S* = 60 J K^−^^1^ mol^−1^, and Δ^‡^ *V* = 25 mL mol^−1^for Δ^‡^ *κ*’ values of (A,D) 0.00 mL mol^−1^ MPa^−1^, (B,E) 0.05 mL mol^−1^ MPa^−1^, and (C,F) 0.10 mL mol^−1^ MPa^−1^.

Aromatic ring flips serve as textbook examples of two‐state chemical exchange with equal populations. They can be investigated by NMR spectroscopy if the chemical shifts between the two sides of the ring differ (Δ*ω* > 0), which they often do [12]. If the exchange rate is slow, *k*_ex _<< Δ*ω*, two separate peaks are observed in the NMR spectrum for the two different positions on either side of the aromatic ring. Increasing the exchange rate (*k*_ex _< Δ*ω*) leads to broadening of these signals, the extent of which depends on the relation of *k*_ex_ to Δω. If the exchange reaches the intermediate regime, where *k*_ex _~ Δ*ω*, signals are maximally broadened and often unobservable. For faster exchange, *k*_ex _> Δ*ω*, one averaged signal is observable at the midpoint between the two individual signals. This signal becomes increasingly sharp as the exchange rate increases, and at the point of very fast exchange *k*_ex _>> Δ*ω*, the signal displays a narrow lineshape. Thereby, the ring flip rates can be studied experimentally by NMR spectroscopy, traditionally using lineshape analysis [7, 13] or exchange spectroscopy [14], and nowadays also by CPMG [15, 17] or *R*_1ρ_ [18, 19] relaxation dispersion experiments in combination with site‐selective isotope labeling to produce suitably isolated ^1^H and ^13^C spins [20, 28]. Ring flips can be studied even when there is no chemical shift difference between the two sides of the ring by measuring relaxation dispersions caused by residual dipolar couplings [29] or, in certain cases, by strong *J* couplings [30]. From temperature‐ and pressure‐dependent studies, one can determine thermodynamic activation parameters, viz., enthalpy (Δ^‡^ *H*) and entropy (Δ^‡^ *S*) [7, 12, 14, 31, 33], volume (Δ^‡^ *V*) [12, 13, 34, 35], and compressibility (Δ^‡^ *κ*) [36]. Our previous study on the aromatic ring of F52 in the B1 domain of staphylococcal protein G (GB1) yielded Δ^‡^ *κ* values indicating that the transition state is liquid‐like, whereas the ground state is solid‐like [36]. Furthermore, all activation parameters listed above were found to be positive, which might be expected since the transition state likely has fewer interactions and is less ordered, more expanded, and more compressible, compared to the ground state. Our study also showed that ring flips can occur with or without a net increase in protein volume; in the latter case, the required local “breathing” around the ring appears to be enabled by migration of void volume through the core to the ring site [36]. This behavior can be appreciated by considering the dependence of the ring flip rate constant (*k*_flip_) on pressure, temperature, and Δ^‡^ *κ* (Figure 1). A positive Δ^‡^ *κ* means implies that pressure has a diminishing effect on *k*_flip_ at higher pressures (Figure 1A–C), approaching a regime where ring flips become effectively pressure‐independent and no longer involve a net volume expansion (Figure 1C). The corresponding diagrams relating Δ^‡^ *G* to pressure and temperature change accordingly (Figure 1D–F). Within the limit of the hydrostatic pressures that can be routinely and safely applied in commercially available high‐pressure NMR tubes (300 MPa or slightly higher), it is possible to measure compressibility coefficients of 0.05 mL mol^−1^ MPa^−1^ or higher with good accuracy (the higher the value, the higher the accuracy), while below 0.05 mL mol^−1^ MPa^−1^, it is getting increasingly difficult to determine the compressibility coefficients (Figure 1).

**FIGURE 2:**
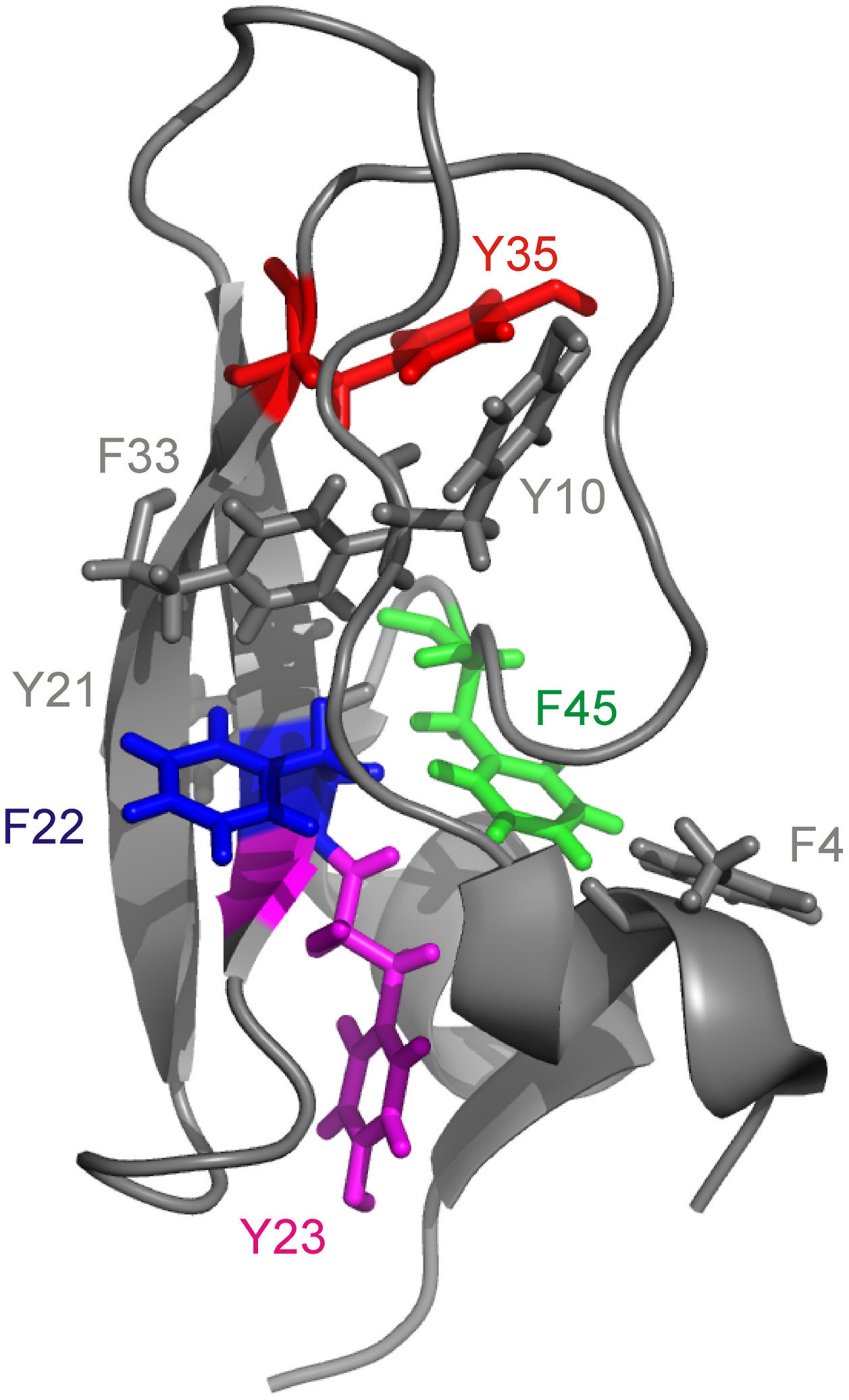
Three‐dimensional structure of BPTI (5pti.pdb) shown as ribbon presentation. Aromatic side chains investigated in this study are shown as sticks in color and are labeled accordingly.

The bovine pancreatic trypsin inhibitor (BPTI) is a small protein of 6.5 kDa exhibiting high solubility and thermodynamic stability against unfolding. It contains three disulfide bridges, four Phe and four Tyr residues, a twisted β‐hairpin, and an α‐helix (Figure 2). Apart from GB1 [12, 18, 29, 36], BPTI is the most extensively studied system for slow ring flips, investigated by NMR spectroscopy [7, 14, 30, 32, 34, 35, 37] as well as molecular dynamics (MD) simulations [38, 40]. Five aromatic residues display slow ring flip rates [30, 32, 38]. Four of them (F22, Y23, Y35, F45) can be studied via chemical shift–mediated exchange broadening, whereas the fifth (Y21) has Δ*ω* = 0 but has been characterized qualitatively. The aromatic rings are located in different parts of the protein and do not interact with each other. Hence, they serve as useful probes of transient conformational changes in different regions of the protein.

Here, we report the activation enthalpy (Δ^‡^ *H*_0_), entropy (Δ^‡^ *S*_0_), volume (Δ^‡^ *V*_0_), and isothermal volume compressibility (Δ^‡^ *κ*’) associated with ring flips involving F22, Y23, Y35, and F45 in BPTI by employing a global analysis of pressure‐ and temperature‐dependent ^13^C relaxation dispersion data for each residue. For the first time, we report activation volumes for F22 and Y23. Moreover, this is the second study determining the isothermal compressibility of ring flips, after establishing this approach on F52 in GB1 [36]. The compressibility constant of activation (Δ^‡^ *κ*’) differs significantly between different residues, ranging from 0.10 (mL mol^−1^ MPa^−1^) for F22 to 0.08 for F45, 0.05 for Y23, and down to 0 for Y35. From Δ^‡^ *κ*’, the compressibility of the transition state (^‡^ *κ*) can be derived. While the ground‐state compressibility is solid‐like [41], ^‡^ *κ* ranges from solid‐like (Y35) to liquid‐like (F22) and correlates negatively with the activation energy of the ring flip. These results show that the packing density of the transition state varies significantly among different structural regions, even for a small protein like BPTI, indicating great variability in the nature of transient conformational changes in proteins.

## 2 Results and Discussion

We performed aromatic ^13^C CPMG and *R*_1ρ_ relaxation dispersion experiments on residues F22, Y23, Y35, and F45 in BPTI (Figure 2) at seven different pressures (0.1, 50, 100, 150, 200, 250, and 300 MPa) and temperatures between 5°C and 65°C. The number of pressure–temperature data sets is limited by the sensitivities of the CPMG and *R*_1ρ_ relaxation dispersion experiments, which depend on the exchange rate constant and the chemical shift difference between the exchanging states [5]. In total, we were able to acquire and analyze 18 data sets for F22, 28 for Y23, 21 for Y35, and 25 for F45 (Figures S1–S4). In the case of F22, the fast NMR exchange regime (resulting in one averaged signal from the two positions of the exchanging nuclei) applies at all pressures and temperatures. In contrast, Y35 is in the slow NMR exchange regime (two signals observed, one from each side of the ring) at all conditions. For Y23 and F45, dispersion experiments were conducted under conditions that covered both exchange regimes, depending on the applied pressure and temperature. The amount of data acquired for each residue is comparable to or greater than that reported in our previous study on residue F52 in GB1, for which 18 relaxation dispersion data sets were analyzed.

In the following, we interpret the exchange as aromatic ring flips. Thus, we fit the ring flip rate constants, *k*_flip_, to the relaxation dispersion curves while keeping the populations fixed, *p*_1 _= *p*_2_ = 0.5, and Δ*δ* fixed at the values measured from HSQC spectra under slow‐exchange conditions or, in the case of F22, determined from CPMG experiments at two static magnetic fields. Potential alternative processes include monomer–oligomer exchange or local conformational exchange associated with disulfide isomerization of C14–C38 [42]. We find it highly unlikely that either of these hypothetical processes could cause the exchange observed for the aromatic side chains because BPTI is known to be fully monomeric below a concentration of 10 mM and to cooperatively form decamers with a lifetime of > 100 min at higher concentrations [43], and the disulfide isomerization involves a minor‐state population of only 1%–5% and exchange rate of > 2500 s^−1^ [42], which cannot reproduce the present dispersion curves.

We initially analyzed the CPMG dispersion data by fitting *k*_flip_ individually at each temperature (Figures S1–S4). In the case of slow exchange, we performed a joint fit of the data from the two sides of each ring position (δ1 and δ2, or ε1 and ε2). Since some of the *R*_1ρ_ dispersion data were acquired under conditions where the exchange rate constants are at the limit of the accessible range, we analyzed them by fitting *k*_flip_ globally to data from all temperatures at a given pressure, while imposing the restrictions *k*_flip_(*T*_high_) > *k*_flip_(*T*_low_) and *R*_2,0_(*T*_high_) ≤ *R*_2,0_(*T*_low_). We fitted the CPMG dispersion data for F22 in two ways: using closed analytical formulae in the form of the Carver–Richards equation [44] or numerical integration of the Bloch–McConnell equations in order to assess limitations of the former to accurately portray symmetric exchange [45] The resulting *k*_flip_ values are identical within errors, but the estimated uncertainties are greater for the numerical fit, particularly at higher pressures (cf. Table S1a,b); for this reason, we used the values fitted using the Carver–Richards equation in the global analysis described below. The flip rate constants determined for the four residues cover a broad range from 5 to 10,000 s^−1^ (Tables S1–S4).

### 2.1 Global Analysis of Temperature‐ and Pressure‐Dependent Flip Rate Constants

**FIGURE 3:**
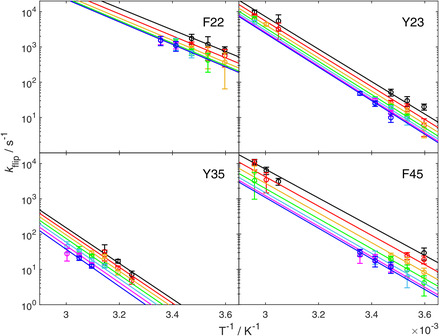
Temperature dependence of flip rates. *k*_flip_ is plotted as a function of 1/T at pressures of 0.1 MPa (black), 50 MPa (red), 100 MPa (yellow), 150 MPa (green), 200 MPa (light blue), 250 MPa (magenta), and 300 MPa (dark blue). The solid lines represent the fit of Equation (2) to the different pressures for global activation parameters of Δ^‡^ *G*_0_, Δ^‡^ *S*_0_, Δ^‡^ *V*_0_, and Δ^‡^ *κ’* at the reference point of 0.1 MPa and 293 K.

Next, we analyzed the flip rate constants obtained at different pressures and temperatures by performing global fits for each of F22, Y23, Y35, and F45 using Equation (2) (Figure 3). We assumed that Δ*δ* is independent of temperature, as confirmed experimentally for Y23, Y35, and F45 under slow‐exchange conditions (Figure S5A). The chemical shifts of Y23 and Y35 exhibit moderate pressure dependence with a slight decrease in Δ*δ* for Y23 with increasing pressure and an increase for Y35 (Figure S5B). Therefore, Y23 and Y35 were fitted using two alternative approaches: either with a constant value of Δδ that was used at all pressures and temperatures, or the actual value of Δδ measured at each pressure under slow‐exchange conditions. The two approaches resulted in identical results within errors (Figure S5C,D).

The global fits to Equation (2) yielded the Gibbs free energy of activation (Δ^‡^ *G*_0_), activation entropy (Δ^‡^ *S*_0_), and activation volume (Δ^‡^ *V*_0_) at the reference point (indicated by the subscript “0”), as well as the volume compressibility of activation (Δ^‡^ *κ*’); see Table 1. The reference point (*p*_0_, *T*_0_) was set to atmospheric pressure (0.1 MPa) and 20°C. The determined values of Δ^‡^ *G*_0_ and Δ^‡^ *S*_0_ for Y23, Y35, and F45 are in good agreement with previous results from relaxation dispersion [32] or lineshape analysis [7, 37]. The ring flip dynamics of F22 have not previously been characterized in terms of thermodynamic parameters of activation, but with the present number of (*p*, *T*) data points, this type of analysis can now be achieved. Similarly, Δ^‡^ *V*_0_ has not previously been reported for Y23. The uncertainty in Δ^‡^ *S*_0_ for F22 and Y35 is greater than for the other residues, since F22 and Y35 could only be studied in the fast and slow‐exchange regimes, respectively, thereby limiting the temperature range yielding suitable data. Prior to the present work, activation volumes of ring flips had only been reported for six residues in three different proteins: in HPr [13], Δ^‡^ *V*_0_ = 27 ± 3 mL mol^−1^ for Y6; in GB1 [12, 36], 26 ± 5 mL mol^−1^ for Y3, 51 ±  11 mL mol^−1^ for Y45, and 29 ± 2 and 28 ± 2 mL mol^−1^ for F52; in BPTI [34, 35], 36 ± 12 and 51 ±  12 mL mol^−1^ for Y35, and 30 ± 6 and 28 ± 5 mL mol^−1^ for F45. The new results for F22 and Y23 in BPTI increase the number of aromatic side chains with experimentally determined activation volumes from six to eight. The Δ^‡^ *V*_0_ values determined here for BPTI are in the same range as those previously reported (Table 1), except that the present value for Y35, Δ^‡^ *V*_0_ = 13 ± 2 mL mol^−1^, is significantly lower than the values from previous studies [34, 35]. We note that the previous results for Y35 differ from one another and exhibit higher uncertainties than the present result, suggesting the apparent differences may not be significant. Taking all available data into account, activation volumes of 20–30 mL mol^−1^ are the most common, with eight out of 13 measurements (including repeats) falling in this range.

**TABLE 1:** Thermodynamic parameters of ring flips in BPTI.^a^

	Δ^‡^ *G*_0_, kJ mol^−1^	Δ^‡^ *S*_0_, J mol^−1^ K^−1^	Δ^‡^ *V*_0_, mL mol^−1^	Δ^‡^ *κ*’, mL mol^−1^ MPa^−1^
F22	52.4 ± 0.5	13 ± 35	22 ± 6	0.10 ± 0.04
Y23	60.7 ± 0.2	85 ± 4	18 ± 3	0.05 ± 0.02
Y35	71.2 ± 0.5	78 ± 24	13 ± 2	0
F45	59.6 ± 0.4	54 ± 9	30 ± 6	0.08 ± 0.04

a Results from global fitting using Equation (2). The reference point is set to (*p*_0_,*T*_0_) = (0.1 MPa, 20°C).

### 2.2 The Volume Compressibility of Activation Differs for Different Rings

**FIGURE 4:**
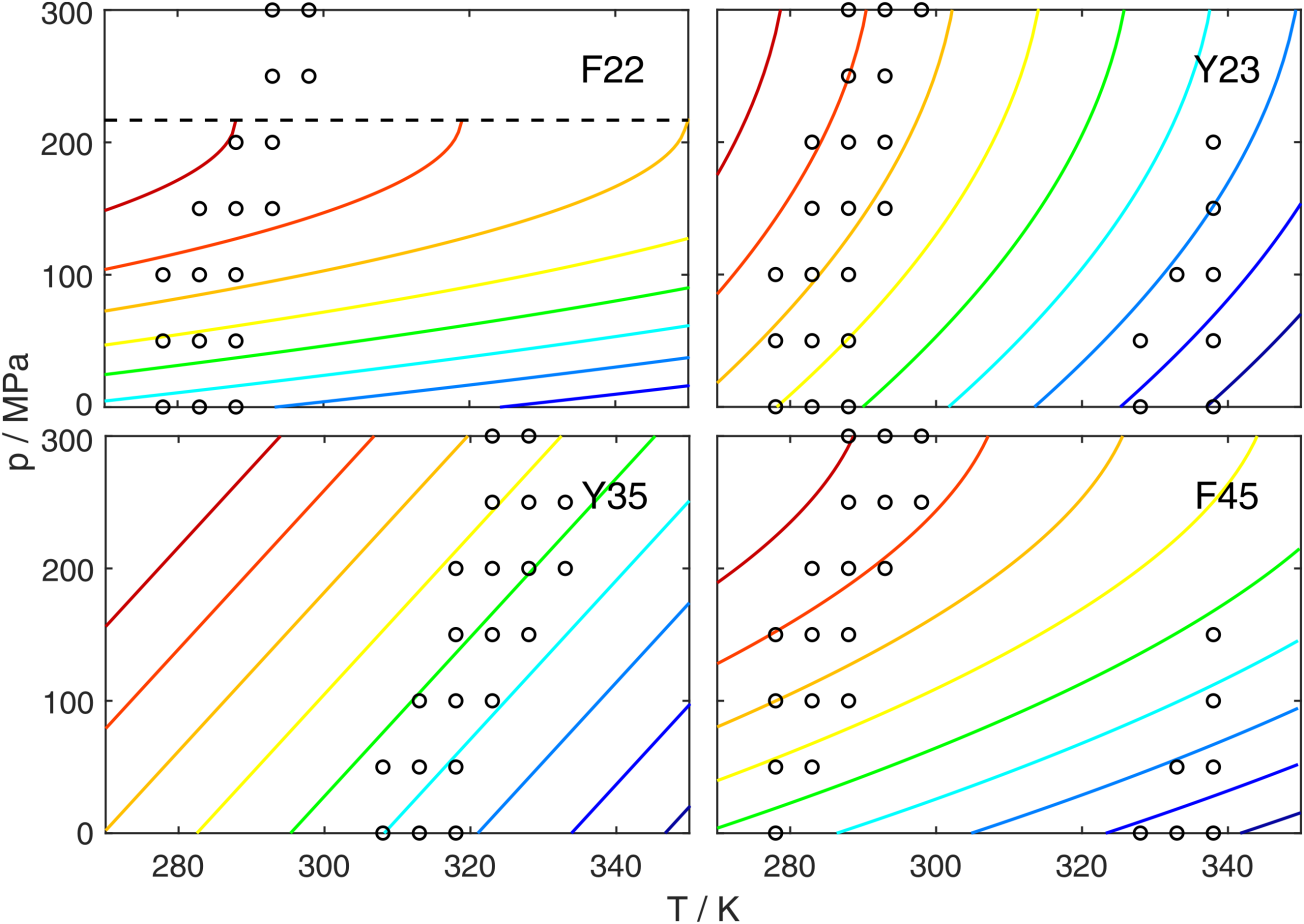
Experimental pressure–temperature phase diagrams of ring flips. Colored lines indicate contours of constant values for the Gibbs free energy of activation, Δ^‡^ *G*, ranging equidistantly (blue to red) from (F22) 52 to 55 kJ mol^−1^, (Y23) 57 to 65 kJ mol^−1^, (Y35) 67 to 75 kJ mol^−1^, and (F45) 57 to 65 kJ mol^−1^. Black circles indicate measured data points, and the dashed line shows the pressure where Δ^‡^ *V* = 0 for F22.

We determined the volume compressibility of activation from the global fits, as previously demonstrated for residue F52 in GB1 [36]. The values of Δ^‡^ *κ*’ for F22 and F45 are (0.10 ± 0.04) and (0.08 ± 0.04) mL mol^−1^ MPa^−1^, respectively. For Y23, Δ^‡^ *κ*’= (0.05 ± 0.02) mL mol^−1^ MPa^−1^ and thus somewhat smaller than the values for the phenylalanines. For these three residues, Δ^‡^ *κ*’ is in the range where the nonlinearity of the pressure dependence is clearly visible and reflected in the data (Figure 4, cf. Figure 1E,F). Furthermore, the nonlinearity is also apparent in the plots of flip rates versus inverse temperature as a decreasing spacing between lines with increasing pressure (Figure 3); that is, the effect of pressure on the flip rates is gradually reduced at higher pressures. The determined compressibility values are comparable to that of F52 in GB1, Δ^‡^ *κ*’ = (0.13 ± 0.02) mL mol^−1^ MPa^−1^. In contrast to the values measured for F22, Y23, and F45, Δ^‡^ *κ*’ is very low for Y35, which is reflected in the equal spacing of the lines fitted for each of the seven pressures (Figure 3). This case is clearly outside the range in which the nonlinearity of the pressure dependence can be accurately determined (cf. Figure 1). Indeed, the best fit yields Δ^‡^ *κ*’ = (0.01 ± 0.02) mL mol^−1^ MPa^−1^ and the *F*‐statistic indicates that it is not suitable to include the compressibility as a parameter; therefore, we performed the global analysis without including Δ^‡^ *κ*’ for this residue. This result does not necessarily mean that Δ^‡^ *κ*’ is zero, but simply that it is too small to be accurately determined, likely between 0 and 0.05 mL mol^−1^ MPa^−1^. From the fitted parameters, four different *p*–*T* diagrams can be generated (Figure 4), three with nonlinear pressure dependence (F22, Y23 and F45) and one with linear pressure dependence (Y35). The experimental data clearly reveal differences between the different residues in their compressibilities. For F22, increasing hydrostatic pressure to 300 MPa brings the system into the regime where further changes in pressure have no effect on the ring flip rate constants (Figures 3A and 4). Nevertheless, ring flips still occur at a rate of  > 10^3^ s^−1^ at room temperature, without overall expansion of the protein.

### 2.3 Estimating the Compressibility and Volume of the Transition States for Ring Flips in BPTI

The isothermal compressibility coefficient of the transition state is given by ^‡^ *κ *= *κ* + Δ^‡^ *κ*, where *κ* refers to the isothermal compressibility coefficient of the ground state. To estimate ^‡^ *κ* from the measured volume compressibility of activation, we make the first‐order approximation Δ^‡^ *κ’*  ≈ *κ*Δ^‡^ *V *+ *V*Δ^‡^ *κ*, where *V* refers to the volume accessible to a given aromatic ring in the ground state and Δ^‡^ *V* is the activation volume determined herein. Considering that Δ^‡^ *V* is the difference in volumes occupied by an aromatic ring in the ground state and in the transition state, the volume of the transition state is given by ^‡^ *V*  = *V* + Δ^‡^ *V*. For each aromatic ring, we calculate *V* based on additively weighted Voronoi tessellation and subsequently calculate ^‡^ *V*, yielding the values listed in Table 2. MD simulations have indicated that the compressibility of BPTI in the ground state is *κ* = 0.23 ± 0.02 GPa^−1^ [41]. Based on these values, we then calculate ^‡^ *κ* and find that it ranges between 0.23 and 1.4 GPa^−1^ (Table 2).

**TABLE 2:** Volumes and compressibilities of ring flips in BPTI.

	*V* a, mL mol^−1^	^‡^ *V* b, mL mol^−1^	Δ^‡^ *κ*, GPa^−1^	^‡^ *κ* c, GPa^−1^
F22	78.0	100	1.2 ± 0.5	1.4 ± 0.5
Y23	70.1	88	0.7 ± 0.3	0.9 ± 0.3
Y35	69.0	82	0	(≥0.2 ± 0.3)
F45	76.2	106	1.0 ± 0.5	1.2 ± 0.5

a Using PDB 5pti.

b ^‡^ *V* = Δ^‡^ *V*_0_–*V*; calculated using Δ^‡^ *V*_0_ from Table 1.

c Calculated using *κ *= 0.23 ± 0.02 GPa^−1^ [41].

The compressibility comprises contributions from protein volume fluctuations and cross‐correlated volume fluctuations of the protein and surrounding water shell [41], but our present work cannot differentiate between these contributions. However, we believe that compressibility measured via ring flips primarily reflects volume fluctuations in the immediate surroundings of the aromatic ring. In principle, *κ* might vary across the structure, as has been inferred from the pressure dependence of NMR order parameters in ubiquitin [46], but we expect these variations to be small compared to the difference in *κ* between the ground and transition states. The variation in *κ* among four different proteins (PGB1, BPTI, ubiquitin, and antifreeze protein) is 12%; the average and standard deviation is (0.21 ± 0.026) GPa^−1^ [41]. Using this variation between proteins as a rough proxy for the variation within a single protein, we expect that the current estimates of ^‡^ *κ* are unlikely to be strongly affected by potential differences in the ground‐state compressibility. This is further supported by the limited pressure dependence of aromatic ^1^H and ^13^C chemical shifts (Figure S6).

The derived isothermal compressibility coefficients of the transition states (Table 2) can be compared to those of various compounds (reported at 1 atm and 25°C [47]). We find that the different transition states in BPTI range from solid‐like to liquid‐like. The lowest value is apparently similar to the ground‐state compressibility of BPTI, i.e., solid‐like, whereas the highest value of 1.4 GPa^−1^ is similar to *κ* of heptane (*κ* = 1.44 GPa^−1^) and the intermediate values of 0.9 and 1.2 GPa^−1^ are similar to *κ* of tetradecane (*κ* = 0.91 GPa^−1^) and nonane (*κ* = 1.18 GPa^−1^); another relevant comparison is benzene, which has a compressibility of 0.966 GPa^−1^. Thus, these values of ^‡^ *κ* suggest that the transition states undergo volume fluctuations similar to liquid alkanes of medium‐ to long‐chain lengths or benzene. For comparison, our previous results obtained for F52 in PGB1 indicated a transition‐state compressibility similar to *κ* of hexane.

### 2.4 The Transition‐State Compressibility Varies Among Different Regions of the BPTI Structure

By mapping the residue‐specific compressibilities of the transition states (^‡^ *κ*) onto the structure of BPTI (Figure 2), it becomes clear that ^‡^ *κ* varies across the structure. At one end of BPTI (at the top in the view of Figure 2), Y35 remains solid‐like at the transition state, whereas Y23 at the other end experiences a semisolid environment, and the central region around F22 and F45 is essentially liquid‐like. Thus, the three different structural regions of BPTI display different behavior at the transition state of the ring flip in terms of retaining or loosening the solid‐like character of the tightly packed ground state. The solid‐like character of the region surrounding Y35 is consistent with the observation that bound water molecules in this area exchange slowly with the bulk solvent [38, 41, 48, 49].

### 2.5 Possible Correlations of the Transition‐State Compressibility and Volume

**FIGURE 5:**
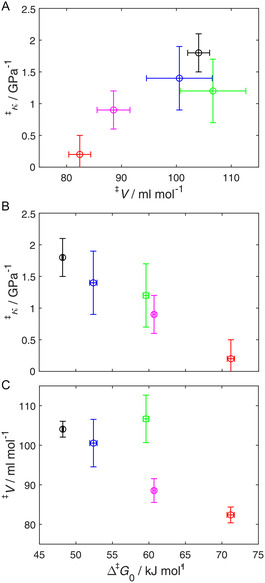
Correlations between fitted values of (A) the compressibility of the transition state, ^‡^ *κ*, and the volume of the transition state, ^‡^ *V*; (B) ^‡^ *κ* and Gibbs free energy of activation, Δ^‡^ *G*_0_; and (C) ^‡^ *V* and Δ^‡^ *G*_0_ (color coding: F22 blue; Y23 magenta; Y35 red; F45 green; and F52 of GB1 [36] black, respectively).

The volume compressibility of activation (Δ^‡^ *κ’*) has now been determined for ring flips of five different aromatic residues in PGB1 and BPTI, with values ranging from near zero to 0.13 mL mol^−1^ MPa^−1^. The corresponding activation volume of these five rings varies between 13 and 30 mL mol^−1^. As stated above, we believe that the observed variation in Δ^‡^ *κ’* mainly arises from differences among the transition states. Therefore, we analyzed the correlations between the transition‐state values ^‡^ *κ*, ^‡^ *V*, and the Gibbs free energy of activation (Δ^‡^ *G*). We observe a roughly linear correlation between ^‡^ *κ* and ^‡^ *V* (Figures 5A and S7), demonstrating that transition states with a larger volume also have a greater compressibility, a result that makes intuitive sense. Furthermore, both ^‡^ *κ* and ^‡^ *V* are inversely correlated with Δ^‡^ *G* (Figures 5B,C and S7), suggesting that ring flips involving high free energy transition barriers occur with less volume expansion and hence relatively lower compressibility of the transition state. In addition, there is a tendency for residues with larger Voronoi volumes in the ground state to have larger Δ^‡^ *V* and ^‡^ *κ* but lower Δ^‡^ *G* (cf. Tables 1 and 2 and Figure S8), suggesting that less densely packed structural regions enable greater volume expansion in the transition state at a lower cost in free energy. However, we note that F45 is an outlier in Figure 5C and, to a lesser extent, also in Figure 5A, which might suggest more complex relationships than the apparent linear trends—for example, compressibility changes not related to volume expansion, as observed at the high‐pressure regime where ring flips can persist without net expansion [36]. These observations should motivate further studies on other proteins to expand the data set on ring flip activation volume and transition‐state compressibility.

## 3 Conclusions

Using a global analysis of pressure‐ and temperature‐dependent ring flip dynamics in BPTI, we have determined the activation parameters of this process, viz., enthalpy (Δ^‡^ *H*_0_), entropy (Δ^‡^ *S*_0_), volume (Δ^‡^ *V*_0_), and isothermal volume compressibility (Δ^‡^ *κ*’) for residues F22, Y23, Y35, and F45. Activation volumes for F22 and Y23 are reported for the first time, as are isothermal volume compressibilities for all four rings. This expands from six to eight the number of residues for which activation volumes have been determined and from one to five the number of isothermal volume compressibilities. Notably, the compressibilities of the transition state in BPTI differ significantly, ranging from solid‐like to liquid‐like, as is typical for folded and unfolded proteins, respectively. These results highlight the different extents of structural fluctuations in a small protein. The isothermal compressibility and, to a lesser extent, the volume of the transition state correlate negatively with the activation energy of the ring flip, showing that the likelihood of ring flip transitions increases with the extent of expansion in the transition state. In summary, the volume and isothermal compressibility of the transition state for ring flips are informative thermodynamic parameters that offer unique structural insights into site‐specific conformational fluctuations in proteins. We believe that extending measurement of ^‡^ *V* and ^‡^ *κ* to a broader range of proteins with diverse structural features will yield a deeper understanding of protein dynamics.

## 4 Experimental Section

### 4.1 NMR Spectroscopy

Aromatic ^13^C L‐TROSY‐CPMG [17] and ^13^C L‐TROSY‐*R*_1ρ_ [19] relaxation dispersion experiments were performed on an 8.5 mM sample of non‐labeled BPTI in 90% H_2_O/10% D_2_O, pH 7.0; thus, the NMR‐active isotope ^13^C was present at the natural abundance of 1.1%. BPTI is fully monomeric below a concentration of 10 mM [43]. BPTI (also known as aprotinin) was purchased from Active Bioscience GmbH. Experiments were performed on a Bruker Avance III 600 NMR spectrometer at a static magnetic field strength of 14.1 T, temperatures between 5°C and 65°C, and hydrostatic pressures between 0.1 and 300 MPa. In addition, experiments were acquired on a Bruker Avance III 800 NMR spectrometer at a static magnetic field strength of 18.8 T, a temperature of 5°C, and a hydrostatic pressure of 0.1 MPa. The exact combinations of temperature and hydrostatic pressure can be found in the Supporting Information (Figures S1–S4). At the chosen conditions, the line broadening *R*_ex_ was measurable with the attainable refocusing field strengths and yielded dispersion profiles with sufficient signal‐to‐noise to enable reliable analysis of activation volume and compressibilty. The spectrometer temperature was calibrated using methanol‐d_4_ following standard protocols [50]. CPMG experiments were recorded with spectral widths of 14.0 ppm (^1^H) and 22.0 ppm (^13^C), and *R*_1ρ_ experiments with spectral widths of 14.0 ppm (^1^H) and 30.0 ppm (^13^C); in both cases, the ^1^H and ^13^C dimensions were sampled by 1024 and 256 points, respectively. The total CPMG relaxation period was 30 ms, and the refocusing frequencies (*ν*_cp_) were 67, 133, 200, 333, 467, 600, and 800 Hz. *R*_1ρ_ experiments were recorded on‐resonance (tilt angle of *θ* = 90°) using a relaxation period of 20 ms and *B*_1_ field strengths of 823, 1005, 1203, 1445, 1971, 2588, 2975, and 4075 Hz. The *B*_1_ field strengths of the spin lock were calibrated by measuring the residual ^1^ *J*_HC_ couplings in an aromatic ^1^H–^13^C HSQC acquired with continuous wave decoupling during acquisition [51]. High‐pressure experiments were performed using commercial 3 and 5 mm ceramic cells [52] (Daedalus Innovations LLC), connected to a home‐built pressure generator, consisting of a water column pressurized by a hand pump. Spectra were processed with NMRPipe [53] and analyzed with PINT [54].

### 4.2 Data Analysis

All relaxation dispersion data were analyzed using fixed populations, *p*_1 _= *p*_2_ = 0.5, and Δ*δ* fixed at the values measured from HSQC spectra under slow‐exchange conditions: 1.53 ppm for Y23ε, 2.21 ppm for Y35ε, 1.90 ppm for F45δ, and 0.49 ppm for F45ε [32]; or, in the case of F22, determined from experiments acquired at two static magnetic fields. The shift difference was assumed to be independent of temperature [7, 13, 32], which was confirmed experimentally for the three residues in slow exchange (Figure S5A). Y23 and Y35 showed some pressure dependence of Δ*δ* (Figure S5B) and were therefore fitted in two ways: either assuming a constant value of Δ*δ* (measured at ambient conditions) that was used at all pressures, or using the Δ*δ* value measured at each pressure under slow‐exchange conditions. CPMG relaxation dispersion data were fitted to the Carver–Richards equation [44] (Equation S1) and using numerical integration of the Bloch–McConnell equations, assuming ideal refocusing pulses in the CPMG train (Equation S2), in the case of fast exchange (F22). In the case of slow exchange (Y23, Y35, and F45), they were fitted to the formula derived by Tollinger et al. [55, 56] (Equation S3). *R*_1ρ_ relaxation dispersion data (Y23 and F45) were fitted to the general equation for symmetric exchange derived by Miloushev and Palmer [57] (Equation S4). The relevant equations were fitted simultaneously to data obtained at different temperatures with the restrictions: *k*_flip_ (*T*_high_) > *k*_flip_ (*T*_low_), *R*_2,0_ (*T*_high_) ≤ *R*_2,0_ (*T*_low_) [12, 36].

Data analysis was performed in MATLAB. Errors in the fitted parameters were estimated using Monte Carlo simulations with 1000 synthetic samples [58]; the reported errors correspond to one standard deviation.

### 4.3 Pressure and Temperature Dependence of Flip Rates

Activation barriers of the ring flips were determined by nonlinear regression of the flip rates, *k*_flip _= *k*_ex_/2, on the temperature *T* using the Eyring equation, parameterized as

(1) $$k_{\text{flip}}=\left(\frac{k_{B}T}{h}\right)\times \exp\left[-\frac{{\text{Δ}}^{‡}G}{RT}\right]$$

where *k*_B_ and *h* denote Boltzmann's and Planck's constants, respectively, and Δ^‡^ *G* is the Gibbs free energy of activation. The pressure and temperature dependence of Δ^‡^ *G* can be obtained by integrating the fundamental equation *d*Δ^‡^ *G* = ‒Δ^‡^ *S* d*T* + Δ^‡^ *V* d*p* from an arbitrarily chosen reference point (*p*_0_, *T*_0_). Assuming that the temperature and pressure dependencies of Δ^‡^ *S* and Δ^‡^ *V* are negligible, we obtain [36]

(2) $$\begin{array}{cc} {\text{Δ}}^{‡}G\left(p,T\right) & \begin{array}{cc} = & {\text{Δ}}^{‡}G_{0}-{\text{Δ}}^{‡}S_{0}\left(T-T_{0}\right) \end{array}\\ & \begin{array}{cc} \;+{\text{Δ}}^{‡}V_{0}\left(p-p_{0}\right) & -\frac{{\text{Δ}}^{‡}\kappa^{'}}{2}{\left(p-p_{0}\right)}^{2} \end{array} \end{array}$$

where Δ^‡^ *κ’ = *Δ^‡^(*κV*) is the volume compressibility of activation, which, as an approximation, can be differentiated into Δ^‡^ *κ’ *≈ *κ*Δ^‡^ *V *+ *V*Δ^‡^ *κ*. The compressibility describes the magnitude of volume fluctuations:

(3) $$\kappa =\frac{‐1}{\left\langle V\right\rangle }{\left(\frac{\partial \left\langle V\right\rangle }{\partial p}\right)}_{T}=\frac{\left\langle {\left(V-\left\langle V\right\rangle \right)}^{2}\right\rangle }{k_{B}T\left\langle V\right\rangle }$$

For Δ^‡^ *κ’* > 0, the pressure–temperature phase diagram of ring flips has a semi‐parabolic shape due to the second‐order pressure dependence of the free energy barrier (Figure 1).

### 4.4 Voronoi Calculations of Accessible Volume

We used the software Voronota version 1.22 to calculate the accessible volume of the aromatic rings by summing the Voronoi volumes of the Cγ, Cδ1, Cδ2, Cε1, Cε2, and Cζ atoms in Phe and Tyr residues and also the Oη atom in Tyr. Calculations were based on the BPTI crystal structure 5pti [59] (1.00 Å resolution).

## Funding

This work was supported by the Vetenskapsrådet (2021‐05591).

## Conflicts of Interest

The authors declare no conflicts of interest.

## Supporting information

Supplementary Material

## Data Availability

The data that support the findings of this study are available from the corresponding author upon reasonable request.
