# Supplementary material for: Pressure‐Dependent Aromatic Ring Flips Reveal Variable Transition‐State Volume and Compressibility Among Structural Regions of BPTI
Source: Chembiochem. 2026 Jun 30;27(13):e70442. doi: 10.1002/cbic.70442 (PMC13316732; doi:10.1002/cbic.70442)
Supplement: Supplementary file 1 — Supplementary Material [file CBIC-27-e70442-s001.pdf]

Pressure Dependent Aromatic Ring Flips Reveal Variable Transition-State Volume and Compressibility Among Structural Regions of BPTI

Matthias Dreydoppel[1], Mikael Akke[2] and Ulrich Weininger[1,*]

[1] Institute of Physics, Biophysics, Martin-Luther-University Halle-Wittenberg, D-06120 Halle (Saale), Germany

[2] Division of Biophysical Chemistry, Center for Molecular Protein Science, Department of Chemistry, Lund University, P.O. Box 124, SE-22100 Lund, Sweden

* Correspondence:

email: ulrich.weininger@physik.uni-halle.de

phone: +49 345 55 28555

fax: +49 345 55 27161

Data analysis

CPMG relaxation dispersion data were fitted to the Carver-Richards equation [1],

$$R_{\mathrm{cp}}^{\mathrm{CR}} = \frac{1}{2}\left(R_{2\mathrm{A}}^{0} + R_{2\mathrm{B}}^{0} + k_{\mathrm{A}} + k_{\mathrm{B}} - \frac{1}{2\tau_{\mathrm{cp}}}\cosh^{-1}[v_{1c}]\right) \tag{1}$$

with

$$v_{1c} = \left[D_{+}\cosh(\eta_{+}) - D_{-}\cosh(\eta_{-})\right]$$

$$D_{\pm} = \frac{1}{2}\left[\pm 1 + \frac{\psi + 2\Delta\omega^{2}}{(\psi^{2} + \xi^{2})^{1/2}}\right]$$

$$\eta_{\pm} = \sqrt{2}\tau_{\mathrm{cp}}\left[\pm\psi + (\psi^{2} + \xi^{2})^{1/2}\right]^{1/2}$$

$$\psi = (-\Delta R_{2}^{0} + k_{\mathrm{A}} - k_{\mathrm{B}})^{2} - \Delta\omega^{2} + 4k_{\mathrm{A}}k_{\mathrm{B}}$$

$$\xi = 2\Delta\omega(-\Delta R_{2}^{0} + k_{\mathrm{A}} - k_{\mathrm{B}})$$

where $R_{2\mathrm{A}}^{0}$ and $R_{2\mathrm{B}}^{0}$are transverse relaxation rates of spins in sites A and B in the absence of exchange, $\Delta R_{2}^{0} = (R_{2\mathrm{B}}^{0} - R_{2\mathrm{A}}^{0})$, $k_{\mathrm{A}} = k_{\mathrm{ex}}\, p_{\mathrm{B}}$, ($k_{\mathrm{B}} = k_{\mathrm{ex}}\, p_{\mathrm{A}}$), $2\tau_{\mathrm{cp}}$ is the time between the CPMG pulses, and $\Delta\omega$ is the chemical shift difference in Hz.

In order to assess limitations of the Carver-Richards equation in the case of symmetric exchange, numerical integration of the Bloch-McConnell equations was performed in accordance with the formula derived by Baldwin [2]:

$$R_{\mathrm{cp}} = R_{\mathrm{cp}}^{\mathrm{CR}} - \frac{1}{4\tau_{\mathrm{cp}}N_{\mathrm{cyc}}}\ln\left(\frac{1+y}{2} + \frac{(1-y)v_{5}}{2(h_{+} + \mathrm{i}h_{-})\sqrt{{v_{1c}}^{2} - 1}}\right) \tag{2}$$

with

$$y = \left[\frac{v_{1c} - \sqrt{{v_{1c}}^{2} - 1}}{v_{1c} + \sqrt{{v_{1c}}^{2} - 1}}\right]^{N_{\mathrm{cyc}}}$$

$$v_{5} = (\Delta R_{2}^{0} + k_{\mathrm{ex}} + i\Delta\omega)v_{1s} - 2v_{4}\sinh(\tau_{\mathrm{cp}}(h_{+} - ih_{-}))$$

$$v_{1s} = \left[D_{+}\sinh(\eta_{+}) - D_{-}\sinh(\eta_{-})\right]$$

$$v_{4} = -\left(2k_{\mathrm{A}} - (\Delta R_{2}^{0} + k_{\mathrm{ex}} - h_{+}) + i(\Delta\omega - h_{-})\right)(h_{-} + \Delta\omega)(\Delta\omega - ih_{+})/({h_{+}}^{2} + {h_{-}}^{2}) + 2\Delta\omega k_{\mathrm{A}}(\Delta\omega - i(\Delta R_{2}^{0} + k_{\mathrm{B}} - k_{\mathrm{A}}))/({h_{+}}^{2} + {h_{-}}^{2})$$

where N_{cyc} is the number of concatenated CPMG elements in the experiment, and $h_{\pm} = (\eta_{\pm}/2\tau_{\mathrm{cp}})$.

In the case of slow exchange, data were fitted to the formula derived by Gutowsky et al. [3] and Tollinger et al. [4]:

$$R_{\mathrm{cp}} = R_{2\mathrm{A}}^{0} + k_{\mathrm{A}} - k_{\mathrm{A}} \frac{\sin(\Delta\omega\tau_{\mathrm{cp}})}{\Delta\omega\tau_{\mathrm{cp}}} \tag{3}$$

$R_{1\rho}$ relaxation dispersion data were fitted to the general equation for symmetric exchange derived by Miloushev & Palmer [5]. In the present case, where the populations $p_A = p_B = 0.5$ and the spin-lock is applied on-resonance with the average signal, the equation reads

$$R_{1\rho} = R_2^0 + \frac{k_{\mathrm{ex}}}{2} - \frac{k_{\mathrm{ex}}}{2}\sqrt{1 - \frac{\Delta\omega^2}{\frac{\omega_{\mathrm{A}}^2\omega_{\mathrm{B}}^2}{\omega_1^2} + k_{\mathrm{ex}}{}^2\left[1 - \frac{\Delta\omega^2}{4}\frac{(\omega_{\mathrm{A}}^2 + \omega_{\mathrm{B}}^2)}{(\omega_{\mathrm{A}}^2\omega_{\mathrm{B}}^2 + k_{\mathrm{ex}}{}^2\omega_1^2)}\right]}} \tag{4}$$

where ω_1 is the spin-lock field strength, and ω_{A} and ω_{B} are the effective field strengths at the resonance frequency of each site (given by $\omega_i^2 = ((\omega_i/2 + \Omega_i)^2 + \omega_1^2$, with Ω_i being the respective Larmor frequency offset from the carrier).

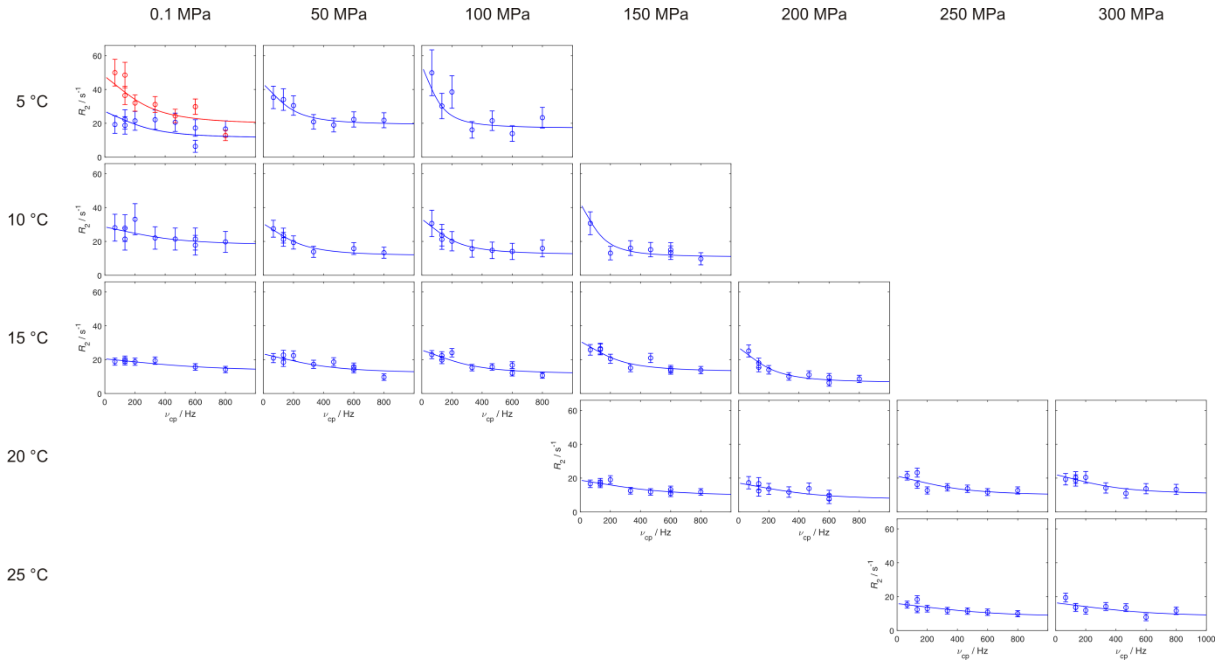

SI Fig 1a: Aromatic ^{13}C CPMG relaxation dispersion profiles for F22ε recorded on an 8.5 mM sample of BPTI at pH 7.0 and static magnetic field strengths of 14.1 T (blue) and 18.8 T (red; only at 278 K and 0.1 MPa). The relaxation dispersions were fitted using the Carver-Richards equation [1] and a fixed population $p_1 = p_2 = 0.5$ and fixed $\Delta\delta$ =0.34 ppm (determined from the fit to data acquired at 5 °C / 0.1 MPa). The determined ring flip rate constants (k_{flip}) are given in SI Table 1. The fitted values of $R_{2,0}$ are 16 ± 3 s^{-1} (5 °C), 12 ± 3 s^{-1} (10 °C), 11 ± 2 s^{-1} (15 °C), 9 ± 1 s^{-1} (20 °C), and 8 ± 1 s^{-1} (25 °C).

`

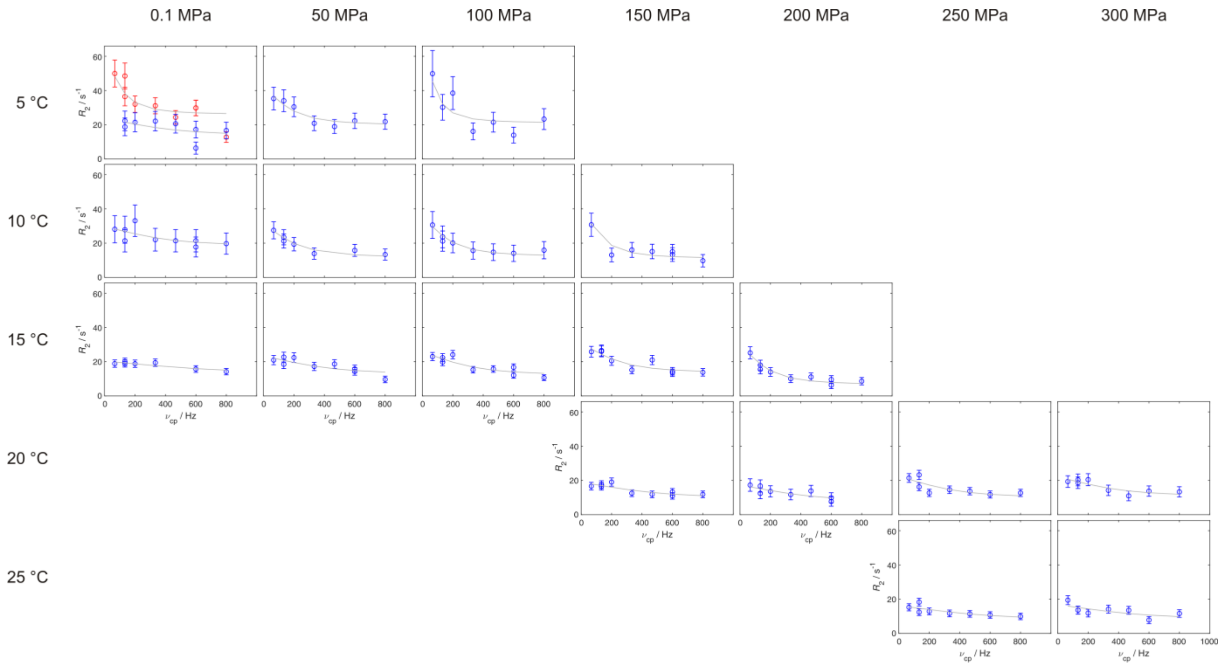

SI Fig 1b: As in Fig. 1a, but the relaxation dispersions were fitted by numerical integration of the Bloch-McConnell equation [2]. The determined ring flip rate constants (k_{flip}) are given in SI Table 1. The fitted values of $R_{2,0}$ are 18 ± 4 s^{-1} (5 °C), 22 ± 4 s^{-1} (10 °C), 14 ± 3 s^{-1} (15 °C), 11 ± 5 s^{-1} (20 °C), and 9 ± 1 s^{-1} (25 °C).

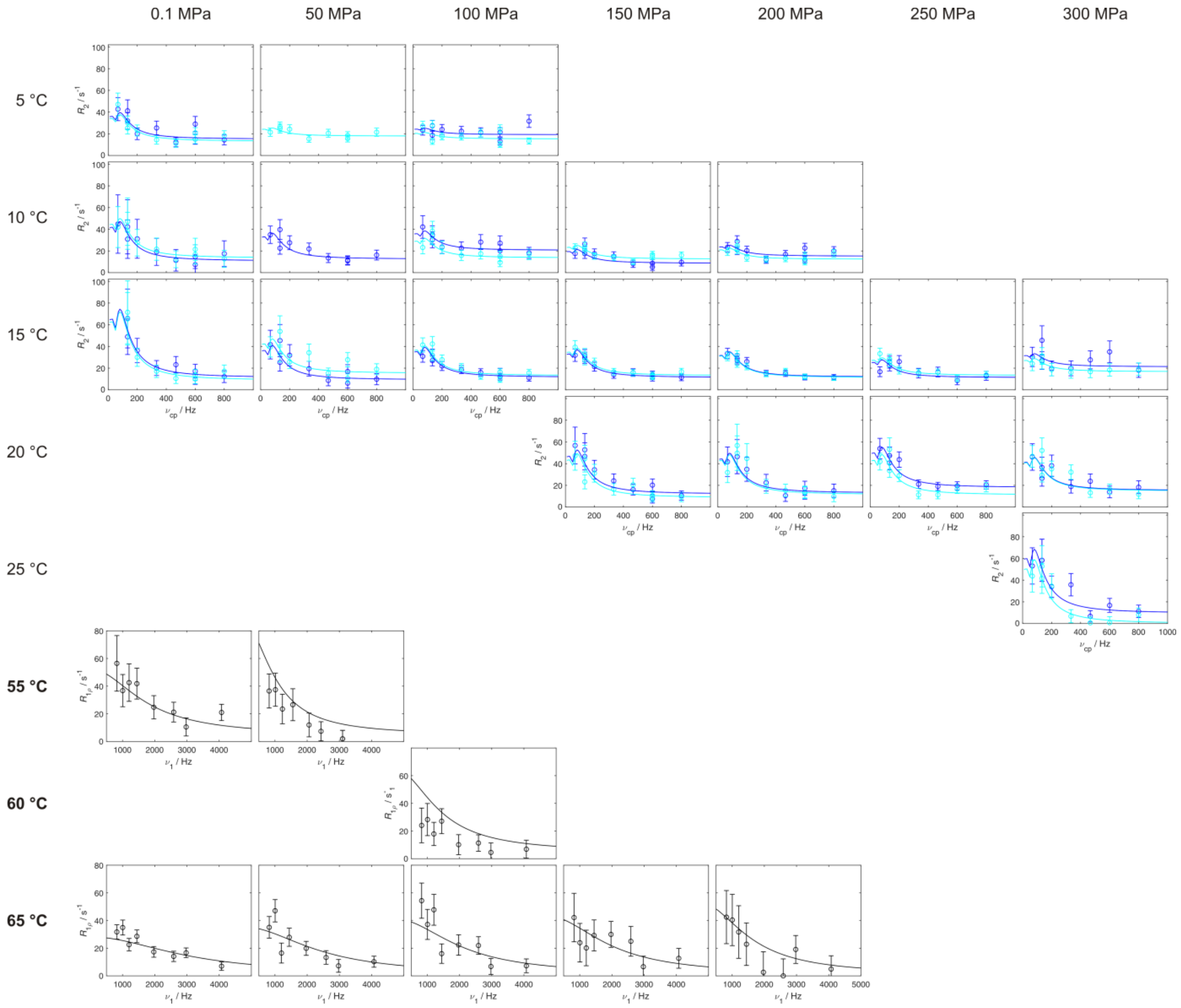

SI Fig 2: Aromatic ^{13}C CPMG (blue) and on-resonance ($\theta > 85°$) $R_{1\rho}$ (black) relaxation dispersion profiles for Y23ε recorded on an 8.5 mM sample of BPTI at pH 7.0 and a static magnetic field strength of 14.1 T. Blue and cyan colours represent datasets for the individual signals of positions 1 and 2, where observable, and black for the single, averaged signal. CPMG relaxation dispersions at a given temperature and pressure were fitted together using the Tollinger formula [3,4] and a fixed population $p_1 = p_2 = 0.5$ and fixed $\Delta\delta = 1.53$ ppm (measured from HSQC spectra under slow-exchange conditions). $R_{1\rho}$ relaxation dispersions (black) where fitted globally using the Miloushev equation [5] with the restrictions: k_{flip} (T_{high}) > k_{flip} (T_{low}), k_{flip} (p_{high}) < k_{flip} (p_{low}), $R_{2,0}$ (T_{high}) ≤ $R_{2,0}$ (T_{low}). The deviations of the fit for 55 °C, 50 MPa and 60 °C, 100 MPa are caused by this global approach, but do not impact further results. When fitting the respective relaxation dispersions individually, the ring flip rates are k_{flip} = (3.6 ± 1.1)·10^3 s^{-1} (55 °C, 50 MPa), and k_{flip} = (1.4 ± 0.5)·10^3 s^{-1} (60 °C, 100 MPa). Determined $R_{2,0}$ are 16 ± 2 s^{-1} (5 °C), 13 ± 4 s^{-1} (10 °C), 14 ± 3 s^{-1} (15 °C), 13 ± 3 s^{-1} (20 °C), 10 ± 3 s^{-1} (25 °C), 5 ± 5 s^{-1} (55 °C), 3 ± 5 s^{-1} (60 °C), and 1 ± 1 s^{-1} (65 °C). The determined ring flip rate constants (k_{flip}) are given in SI Table 2.

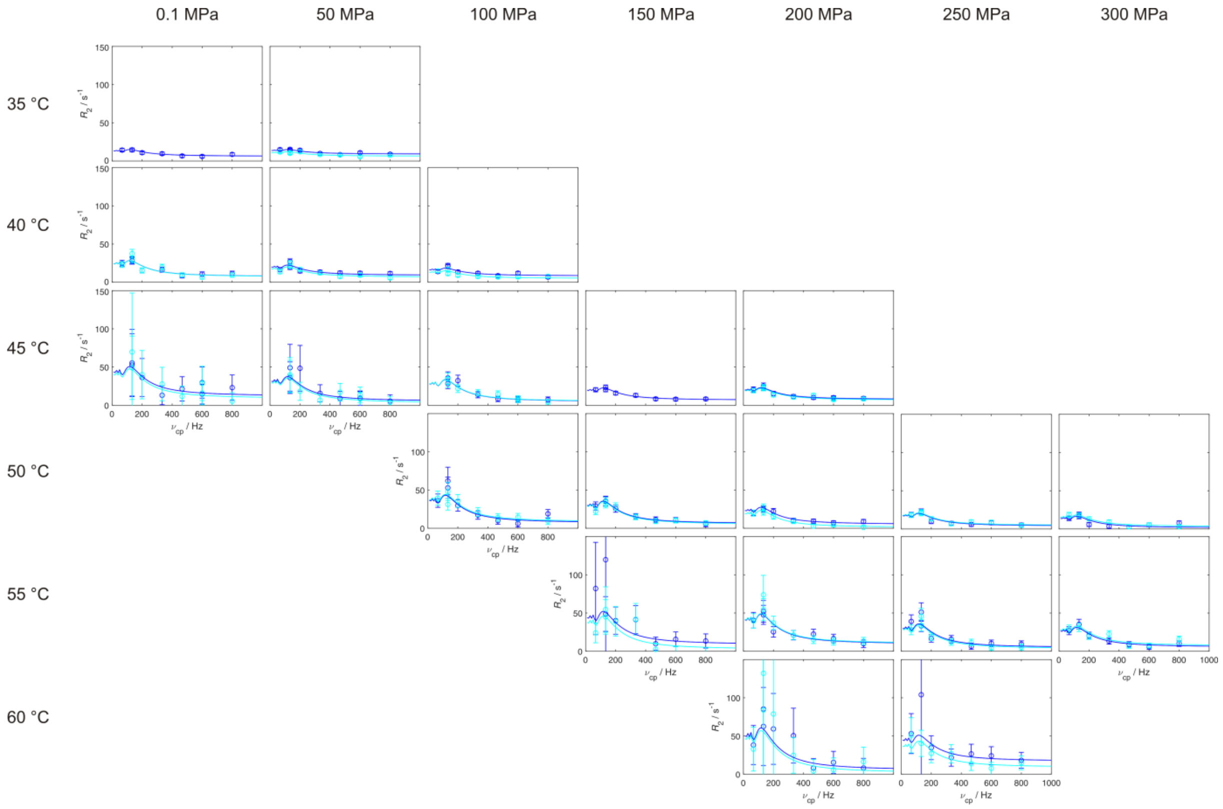

SI Fig 3: Aromatic ^{13}C CPMG relaxation dispersion profiles for Y35ε recorded on an 8.5 mM sample of BPTI at pH 7.0 and a static magnetic field strength of 14.1 T. Blue and cyan colours represent datasets for the individual signals of positions 1 and 2, respectively. CPMG relaxation dispersions at a given temperature and pressure were fitted together using the Tollinger formula [3,4] and a fixed population $p_1 = p_2 = 0.5$ and fixed $\Delta\delta = 2.21$ ppm (measured from HSQC spectra under slow-exchange conditions). Determined $R_{2,0}$ are 7 ± 2 s^{-1} (35 °C), 8 ± 1 s^{-1} (40 °C), 7 ± 3 s^{-1} (45 °C), 5 ± 2 s^{-1} (50 °C), 7 ± 3 s^{-1} (55 °C), and 6 ± 3 s^{-1} (60 °C). The determined ring flip rate constants (k_{flip}) are given in SI Table 3.

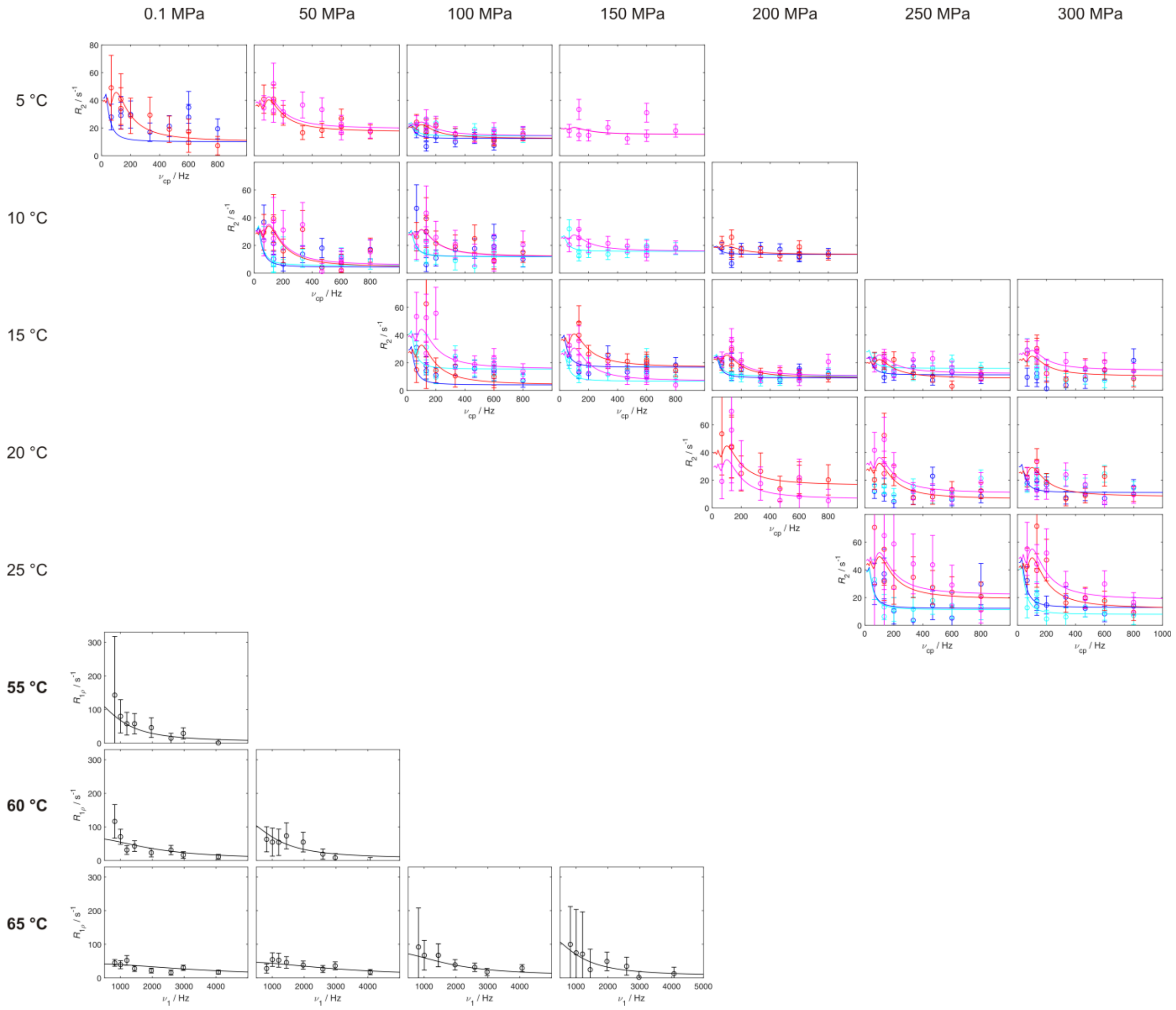

SI Fig 4: Aromatic ^{13}C CPMG and on-resonance ($\theta > 85°$) $R_{1\rho}$ relaxation dispersion profiles for F45ε and F45δ (red and blue), and on-resonance ($\theta > 85°$) $R_{1\rho}$ relaxation dispersion profiles for F45δ (black) recorded on an 8.5 mM sample of BPTI at pH 7.0 and a static magnetic field strength of 14.1 T. Blue and cyan colours represent datasets for the individual signals of positions ε1 and ε2, respectively, red and magenta for δ1 and δ2, and black for the single, averaged signal of δ*. CPMG relaxation dispersions at a given temperature and pressure were fitted together using the Tollinger formula [3,4] and a fixed population $p_1 = p_2 = 0.5$ and fixed values of $\Delta\delta = 1.90$ (for δ) and 0.49 (for ε) ppm (measured from HSQC spectra under slow-exchange conditions). $R_{1\rho}$ relaxation dispersions (black) where fitted globally using the Miloushev equation [5] with the restrictions: k_{flip} (T_{high}) > k_{flip} (T_{low}), k_{flip} (p_{high}) < k_{flip} (p_{low}), $R_{2,0}$ (T_{high}) ≤ $R_{2,0}$ (T_{low}). Determined $R_{2,0}$ are 13 ± 3 s^{-1} (5 °C), 13 ± 4 s^{-1} (10 °C), 13 ± 2 s^{-1} (15 °C), 11 ± 2 s^{-1} (20 °C, 25 °C) for F45ε; and 15 ± 3 s^{-1} (5 °C), 11 ± 5 s^{-1} (10 °C), 11 ± 4 s^{-1} (15 °C), 10 ± 4 s^{-1} (20 °C), 16 ± 4 s^{-1} (25 °C), 4 ± 4 s^{-1} (55 °C), 3 ± 5 s^{-1} (60 °C), and 2 ± 4 s^{-1} (65 °C) for F45δ. The determined ring flip rate constants (k_{flip}) are given in SI Table 4.

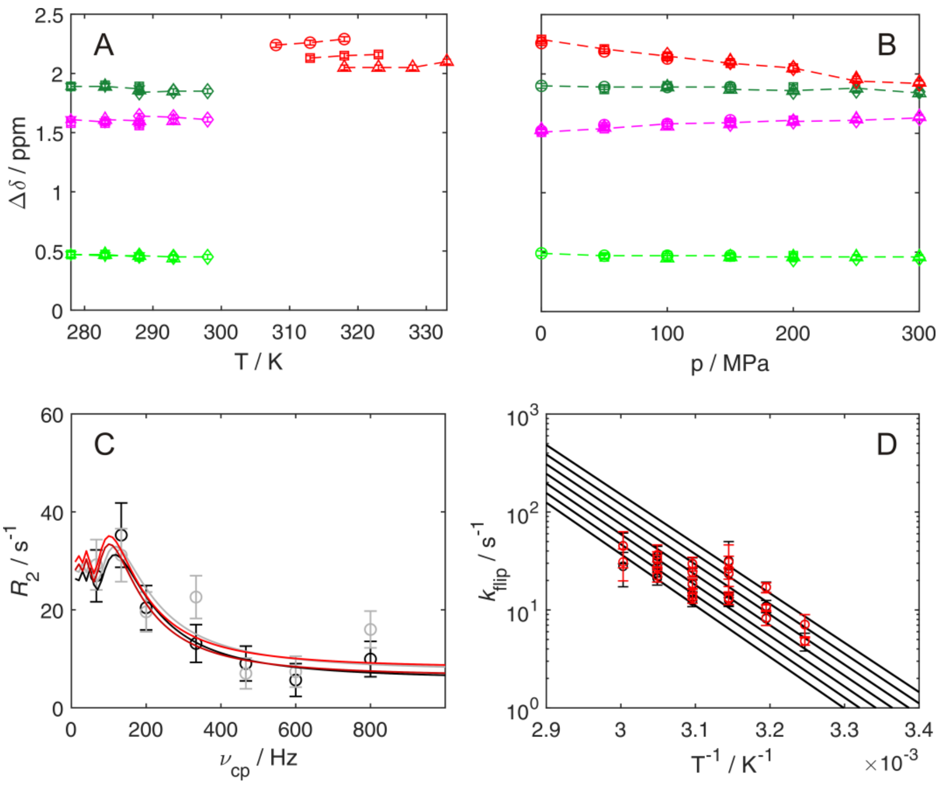

SI Fig 5: (A) Temperature and (B) pressure dependence of the chemical shift difference ($\Delta\delta$) between the two sides of the ring (Y23 magenta, Y35 red, F45δ dark green and F45ε light green; A) circles 0.1 MPa, squares 100 MPa, hexagrams 150 MPa, triangles 200 MPa, diamonds 300 MPa; B) for Y23 and F45: circles 5 °C, squares 10 °C, triangles 15 °C, diamonds 20 °C; for Y35: circles 40 °C, squares 45 °C, triangles 50 °C, diamonds 55 °C). $\Delta\delta$ were derived from $^1H^{13}C$ HSQC spectra under slow exchange conditions. (C) Aromatic ^{13}C CPMG relaxation dispersion profile for Y35ε, recorded at 55 °C and 300 MPa. Black and grey circles represent data for the individual signals of positions 1 and 2, respectively. The data was fitted using the Tollinger formula [3,4] and a fixed population $p_1 = p_2 = 0.5$, and fixed $\Delta\delta = 2.21$ ppm (black, as in SI Fig. 3), or $\Delta\delta = 1.91$ ppm (red). (D) k_{flip} is plotted as a function of 1/T at different pressures. k_{flip} values determined from CPMG relaxation dispersions using either $\Delta\delta = 2.21$ ppm (black) or individual $\Delta\delta$ from the respective HSQC spectra under slow-exchange conditions (red). Solid lines represent the fit of the global activation parameters of eq. (2) of the main text to the different pressures, as in Fig. 3 of the main text. Fiotting data using individual $\Delta\delta$ (red) results in: $\Delta^{\ddagger}G_0 = 72.0 \pm 0.4$ kJ mol^{-1}, $\Delta^{\ddagger}S_0 = 103 \pm 22$ J mol^{-1} K^{-1}, $\Delta^{\ddagger}V_0 = 13 \pm 2$ mL mol^{-1} and $\Delta^{\ddagger}\kappa' = 0$ mL mol^{-1} MPa^{-1}; these results should be compared with those obtained using a fixed value of $\Delta\delta = 2.21$ ppm: $\Delta^{\ddagger}G_0 = 71.2 \pm 0.5$ kJ mol^{-1}, $\Delta^{\ddagger}S_0 = 78 \pm 24$ J mol^{-1} K^{-1}, $\Delta^{\ddagger}V_0 = 13 \pm 2$ mL mol^{-1} and $\Delta^{\ddagger}\kappa' = 0$ mL mol^{-1} MPa^{-1}.

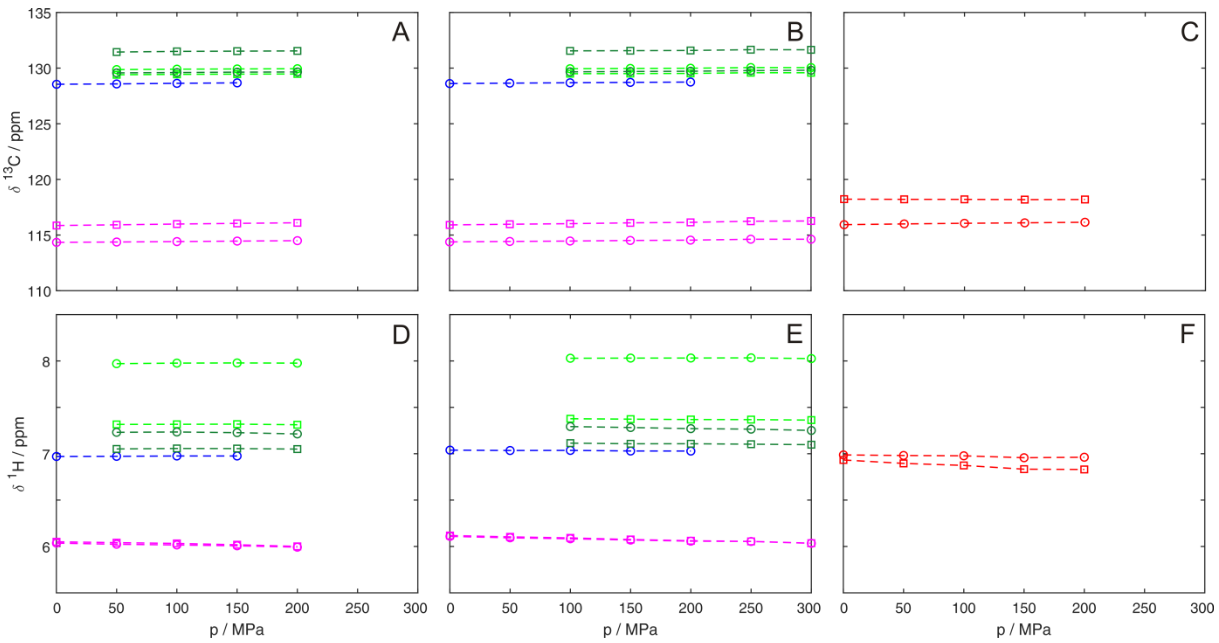

SI Fig 6: Pressure dependence of absolute ^{13}C and ^{1}H chemical shifts at temperatures of 10 °C (A, D), 15 °C (B, E), and 45 °C (C, F). Color coding: F22ε blue, Y23ε magenta, Y35ε red, F45δ dark green, and F45ε light green. Circles and squares depict data from the signals of positions 1 and 2, respectively, except for F22, where only one averaged signal is observed. In the case of Y23ε, $\Delta\delta$ (^{1}H) = 0, resulting in a single line in panels D and E.

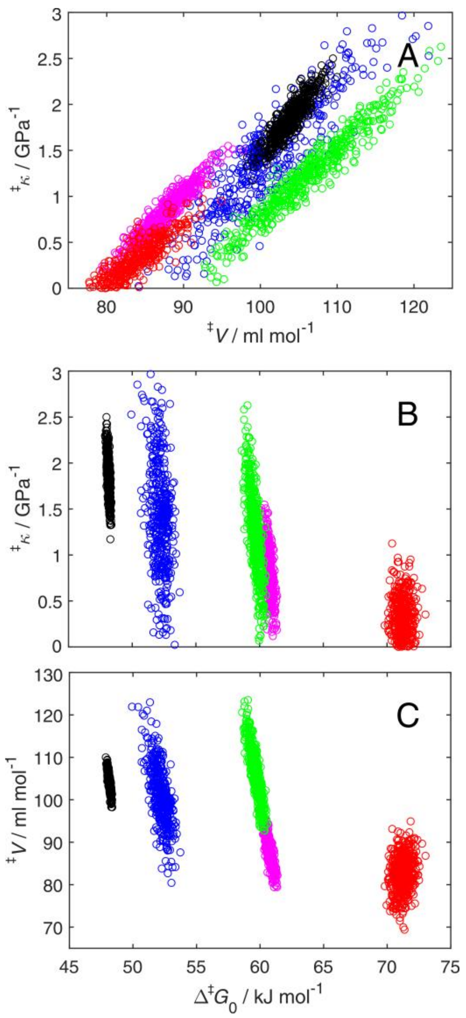

SI Fig 7: Correlations between the fitted values of (A) the compressibility of the transition state, ${}^{\ddagger}\kappa$, and volume of the transition state, ${}^{\ddagger}V$, (B) ${}^{\ddagger}\kappa$ and Gibbs free energy of activation, $\Delta^{\ddagger}G_0$, and (C) ${}^{\ddagger}V$ and $\Delta^{\ddagger}G_0$. Color coding: F22 blue; Y23 magenta; Y35 red; F45 green; and F52 of GB1 [6] black. For each residue, an ensemble of 500 fitted parameter sets was generated by Monte Carlo simulations.

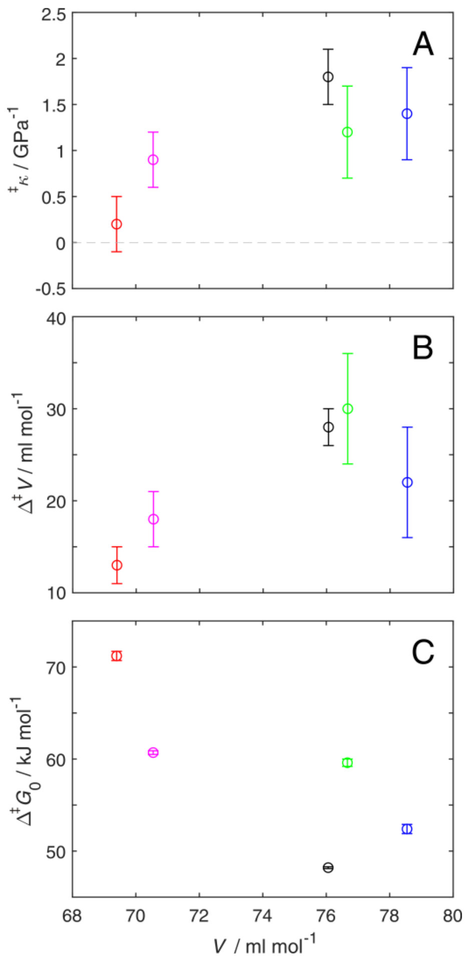

SI Fig 8: Correlations between the Voronoi volumes, *V*, of the ground state and activation parameters. (A) The compressibility of the transition state, $^{‡}\kappa$, plotted vs *V*. (B) The volume difference between the ground and transition states, $\Delta^{‡}V$, plotted vs *V*. (C) The Gibbs free energy of activation, $\Delta^{‡}G_0$, plotted vs *V*. Color coding: F22 blue; Y23 magenta; Y35 red; F45 green; and F52 of GB1 [6] black. The Voronoi volumes of aromatic rings in BPTI were calculated using the PDB structure 5pti [7].

SI Table 1a. Ring flip rates for F22 at different pressures and temperatures obtained using CPMG relaxation dispersion experiments and parameter optimization using the Carver-Richards equation.

k_{flip} (s^{-1})					
p (MPa)	5 °C	10 °C	15 °C	20 °C	25 °C
0.1	820 ± 190	1180 ± 770	1740 ± 540		
50	560 ± 290	690 ± 280	1130 ± 250		
100	370 ± 310	630 ± 390	910 ± 160		
150		420 ± 230	730 ± 160	1360 ± 300	
200			630 ± 140	1290 ± 490	
250				1120 ± 240	1590 ± 420
300				1090 ± 340	1530 ± 450

The relaxation dispersions were fitted using the Carver-Richards equation [1] and a fixed population $p_1 = p_2 = 0.5$ and fixed $\Delta\delta = 0.34$ ppm (determined from the fit to data acquired at 5 °C / 0.1 MPa). Determined $R_{2,0}$ values are 16 ± 3 s^{-1} (5 °C), 12 ± 3 s^{-1} (10 °C), 11 ± 2 s^{-1} (15 °C), 9 ± 1 s^{-1} (20 °C), and 8 ± 1 s^{-1} (25 °C).

SI Table 1b. Ring flip rates for F22 at different pressures and temperatures obtained using CPMG relaxation dispersion experiments and parameter optimization using numerical integration of the Bloch-McConnell equation.

k_{flip} (s^{-1})					
p (MPa)	5 °C	10 °C	15 °C	20 °C	25 °C
0.1	790 ± 690	1100 ± 530	1760 ± 320		
50	570 ± 160	650 ± 360	1170 ± 400		
100	260 ± 350	550 ± 240	920 ± 250		
150		430 ± 360	730 ± 180	1340 ± 250	
200			540 ± 240	1270 ± 400	
250				1000 ± 1510	1510 ± 370
300				1090 ± 1000	1470 ± 1550

The relaxation dispersions were fitted by numerical integration of the Bloch-McConnell equation [2] using a fixed population $p_1 = p_2 = 0.5$ and fixed $\Delta\delta = 0.34$ ppm (determined from the fit to data acquired at 5 °C / 0.1 MPa). Determined $R_{2,0}$ values are 18 ± 4 s^{-1} (5 °C), 22 ± 4 s^{-1} (10 °C), 14 ± 3 s^{-1} (15 °C), 11 ± 5 s^{-1} (20 °C), and 9 ± 1 s^{-1} (25 °C).

SI Table 2a. Ring flip rates for Y23 at different pressures and temperatures using CPMG relaxation dispersion.

k_{flip} (s^{-1}) [a]					
p (MPa)	5 °C	10 °C	15 °C	20 °C	25 °C
0.1	20.1 ± 4.0	29.9 ± 9.3	51.9 ± 11.5		
50	6.1 ± 3.2	19.7 ± 4.8	26.0 ± 4.7		
100	4.9 ± 2.2	14.7 ± 3.7	22.6 ± 2.9		
150		10.4 ± 2.2	20.9 ± 2.6	33.4 ± 5.2	
200		8.3 ± 2.2	19.1 ± 2.1	30.2 ± 6.4	
250			13.4 ± 2.6	30.6 ± 3.9	
300			9.9 ± 2.7	25.1 ± 4.5	48.4 ± 5.8

[a] from joint fit of resolved signals of the ε position. CPMG relaxation dispersions were fitted using the Tollinger formula [3,4] and a fixed population $p_1 = p_2 = 0.5$ and fixed $\Delta\delta = 1.53$ ppm (measured from HSQC spectra under slow-exchange conditions). Determined $R_{2,0}$ are 16 ± 2 s^{-1} (5 °C), 13 ± 4 s^{-1} (10 °C), 14 ± 3 s^{-1} (15 °C), 13 ± 3 s^{-1} (20 °C), and 10 ± 3 s^{-1} (25 °C).

SI Table 2b. Ring flip rates for Y23 at different pressures and temperatures using $R_{1\rho}$ relaxation dispersion.

k_{flip} (10^3 s^{-1})			
p (MPa)	55 °C	60 °C	65 °C
0.1	5.5 ± 2.7		9.6 ± 1.5
50	3.2 ± 1.5		7.6 ± 1.0
100		4.4 ± 0.4	6.5 ± 0.9
150			6.2 ± 1.1
200			5.1 ± 0.7

$R_{1\rho}$ relaxation dispersions were fitted globally using the Miloushev equation [5] and a fixed population $p_1 = p_2 = 0.5$ and fixed $\Delta\delta = 1.53$ ppm (measured from HSQC spectra under slow-exchange conditions), with the restrictions: k_{flip} (T_{high}) > k_{flip} (T_{low}), k_{flip} (p_{high}) < k_{flip} (p_{low}), $R_{2,0}$ (T_{high}) ≤ $R_{2,0}$ (T_{low}). Determined $R_{2,0}$ are 5 ± 5 s^{-1} (55 °C), 3 ± 5 s^{-1} (60 °C), and 1 ± 1 s^{-1} (65 °C).

SI Table 3. Ring flip rates for Y35 at different pressures and temperatures using CPMG relaxation dispersion.

	k_{flip} (s^{-1}) [a]					
p (MPa)	35 °C	40 °C	45 °C	50 °C	55 °C	60 °C
0.1	7.1 ± 1.8	17.2 ± 2.1	31.7 ± 18.2			
50	4.8 ± 1.0	11.0 ± 1.5	26.4 ± 9.0			
100		8.2 ± 1.2	23.5 ± 2.9	29.5 ± 4.5		
150			13.0 ± 2.0	24.1 ± 2.5	35.5 ± 9.1	
200			12.5 ± 1.7	18.2 ± 2.0	32.0 ± 5.1	45.3 ± 15.7
250				13.6 ± 1.8	25.4 ± 3.6	28.0 ± 10.7
300				12.4 ± 1.6	21.0 ± 3.1	

[a] from joint fit of resolved signals of the ε position.

CPMG relaxation dispersions were fitted using the Tollinger formula [3,4] and a fixed population $p_1 = p_2 = 0.5$ and fixed $\Delta\delta = 2.21$ ppm (measured from HSQC spectra under slow-exchange conditions). Determined $R_{2,0}$ are 7 ± 2 s^{-1} (35 °C), 8 ± 1 s^{-1} (40 °C), 7 ± 3 s^{-1} (45 °C), 5 ± 2 s^{-1} (50 °C), 7 ± 3 s^{-1} (55 °C), and 6 ± 3 s^{-1} (60 °C).

SI Table 4a. Ring flip rates for F45 at different pressures and temperatures using CPMG relaxation dispersion.

k_{flip} (s^{-1}) [a]					
p (MPa)	5 °C	10 °C	15 °C	20 °C	25 °C
0.1	29.3 ± 11.0				
50	19.1 ± 4.9	24.5 ± 5.5			
100	8.3 ± 2.6	16.1 ± 5.4	23.7 ± 5.3		
150	4.2 ± 2.4	9.7 ± 3.6	19.8 ± 3.7		
200		6.0 ± 3.0	13.5 ± 2.5	23.5 ± 9.6	
250			11.2 ± 2.8	21.2 ± 5.1	25.3 ± 10.5
300			11.9 ± 4.2	17.4 ± 5.7	30.3 ± 6.6

[a] from joint fit of resolved signals of the δ position together with signals of the ε position. CPMG relaxation dispersions were fitted using the Tollinger formula [3,4] and a fixed population $p_1 = p_2 = 0.5$ and fixed values of $\Delta\delta = 1.90$ (for δ) and 0.49 (for ε) ppm (measured from HSQC spectra under slow-exchange conditions). Determined $R_{2,0}$ are 13 ± 3 s^{-1} (5 °C), 13 ± 4 s^{-1} (10 °C), 13 ± 2 s^{-1} (15 °C), 11 ± 2 s^{-1} (20 °C, 25 °C) for F45ε; and 15 ± 3 s^{-1} (5 °C), 11 ± 5 s^{-1} (10 °C), 11 ± 4 s^{-1} (15 °C), 10 ± 4 s^{-1} (20 °C), and 16 ± 4 s^{-1} (25 °C) for F45δ.

SI Table 4b. Ring flip rates for F45 at different pressures and temperatures using $R_{1\rho}$ relaxation dispersion.

k_{flip} (10^3 s^{-1}) [b]			
p (MPa)	55 °C	60 °C	65 °C
0.1	3.1 ± 0.7	6.4 ± 1.5	11.1 ± 2.0
50		3.5 ± 2.0	9.7 ± 3.1
100			5.7 ± 2.7
150			3.3 ± 2.3

[b] from fit of the averaged signal of the δ position

$R_{1\rho}$ relaxation dispersions (black) where fitted all together using the Miloushev equation [5] and a fixed population $p_1 = p_2 = 0.5$ and fixed values of $\Delta\delta = 1.90$ (for δ) and $\Delta\delta = 0.49$ (for ε) ppm (measured from HSQC spectra under slow-exchange conditions), with the restrictions: k_{flip} (T_{high}) > k_{flip} (T_{low}), k_{flip} (p_{high}) < k_{flip} (p_{low}), $R_{2,0}$ (T_{high}) ≤ $R_{2,0}$ (T_{low}). Determined $R_{2,0}$ are 4 ± 4 s^{-1} (55 °C), 3 ± 5 s^{-1} (60 °C), and 2 ± 4 s^{-1} (65 °C).

References:

[1] J. P. Carver, R. E. Richards, *J. Magn. Reson.* **1972**, *6* (1), 89-105.

[2] A. J. Baldwin, *J. Magn. Reson.* **2014,** *244*, 114-124.

[3] H. S. Gutowsky, R. L. Vold, E. J. Wells, *J. Chem. Phys.* **1965**, *43*, 4107–4125.

[4] M. Tollinger, N. R. Skrynnikov, F. A. A. Mulder, J. D. Forman-Kay, L. E. Kay, *J. Am. Chem. Soc.* **2001,** *123* (46), 11341-11352.

[5] V. Z. Miloushev, A. G. Palmer, *J. Magn. Reson.* **2005,** *177* (2), 221-227.

[6] M. Dreydoppel, B. Dorn, K. Modig, M. Akke, U. Weininger, *Jacs Au* **2021,** *1* (6), 833-842

[7] A. Wlodawer, J. Walter, R. Huber, L. Sjolin, *J. Mol. Biol.* **1984**, *180*, 301-329.
